# Substantial Improvement of Color-Rendering Properties of Conventional White LEDs Using Remote-Type Red Quantum-Dot Caps

**DOI:** 10.3390/nano12071097

**Published:** 2022-03-27

**Authors:** Gi Jung Lee, Seung Chan Hong, Jung-Gyun Lee, Jae-Hyeon Ko, Taehee Park, Young Wook Ko, Sergey Lushnikov

**Affiliations:** 1 School of Nano Convergence Technology, Nano Convergence Technology Center, Hallym University, Chuncheon 24252, Korea; 42772@hallym.ac.kr (G.J.L.); 43193@hallym.ac.kr (S.C.H.); wjdrbsdl97@hallym.ac.kr (J.-G.L.); 2 GLVISION Co., Ltd., Room 1034, 9-36 Supuran-gil, Siheung-si 14986, Korea; thpark@glvision.co.kr (T.P.); ywko@glvision.co.kr (Y.W.K.); 3 Department of Physics of Dielectrics and Semiconductors, Ioffe Institute, Politekhnicheskaya 26, 194021 Saint Petersburg, Russia; sergey.lushnikov@mail.ioffe.ru

**Keywords:** quantum dot, LED, color rendering index, remote, quantum-dot cap

## Abstract

A new type of remote red quantum-dot (QD) component was designed and fabricated to improve the color-rendering properties of conventional white LED (light-emitting diode) lightings. Based on an optical simulation, the rectangular cavity-type QD cap was designed with an opening window on the top surface. Red QD caps were fabricated using a typical injection molding technique and CdSe/ZnS QDs with a core/shell structure whose average size was ~6 nm. Red QD caps were applied to conventional 6-inch, 15-W white LED downlighting consisting of 72 LEDs arrayed concentrically. The red QD caps placed over white LEDs enhanced the red components in the long-wavelength range resulting in the increase of the color rendering index (CRI) from 82.9 to 94.5. The correlated color temperature was tuned easily in a wide range by adopting various configurations consisting of different QD caps. The spatial and angular homogeneities were secured on the emitting area because QD caps placed over the white LEDs did not exhibit any substantial optical path length difference. The present study demonstrates that adopting QD caps in conventional LED lightings provides a flexible and efficient method to realize a high color-rendering property and to adjust correlated color temperature appropriately for a specific application.

## 1. Introduction

The invention of blue light-emitting diodes (LEDs) based on GaN in the 1990s has been revolutionizing current lighting technologies. The combination of blue LEDs and color conversion materials has been used to generate white light for general lighting and display backlight applications [1]. The most conventional color conversion material is Ce-doped yttrium aluminum garnet (YAG, Y_3_Al_5_O_12_), which converts part of the blue light into yellow light via Stokes shift [2]. This approach is very cost-effective and efficient, while the insufficient deep red component makes the color-rendering property of this white LED worse than other typical light sources, such as incandescent lamps or fluorescent lamps. During the COVID-19 period, people have spent a much longer time in buildings or houses; thus, indoor activity has become more important than before. As a result, the color rendering characteristics of general lighting have arisen as one of the important factors that determine quality of life in civil architecture.

Adopting green and red phosphors instead of a single-component yellow phosphor in the white LED is one approach to secure good color rendering properties [3]. In this case, the red phosphor absorbs blue and green photons and then emits red photons in the long-wavelength range resulting in a high color rendering index (CRI). CRI is a standard metric whereby the color appearance of objects can be estimated in comparison with standard light sources [4]. Another way to increase the CRI of white LEDs is to add red quantum dots (QDs) as a color conversion material [5,6]. QD is a nanometer-sized semiconductor and its quantum confinement effect plays an important role in its emitting properties [7,8,9]. The emitting wavelength of QD increases with decreasing size; thus, it is easy to tune the emitting wavelength by only changing the dimension of QD. QD has attracted great attention due to its easy processibility, broad color tunability, high photoluminescent quantum efficiency, and flexibility in its application to the lighting technology. Surface treatment can easily be conducted for reliability, high quantum efficiency, and homogeneous dispersion in various matrices. Due to the high color purity, QDs have penetrated the display market, mainly in backlights for liquid crystal displays [10,11,12,13,14,15,16].

QD can be mixed with phosphors and coated over the blue LEDs. However, QD is vulnerable to the high temperature generated from the LED chips. Various remote QD components have been adopted to overcome this degradation problem. Nowadays, QD films are widely used in backlights for liquid crystal displays to increase the color gamut. Other types of remote QD components have also been studied and applied to various lighting structures [17,18,19,20,21,22,23]. These efforts showed that the color rendering characteristics can be improved substantially by adopting QD materials [24]. From the early stage of research of QD applications in lightings and displays, QD-polymer or QD-glass composites have been widely investigated by various groups [25,26,27,28,29,30,31,32,33,34,35,36,37,38]. Successful fabrication of large-size QD films accelerated their applications in display backlights and general lighting. However, the optical path length difference in the QD component sometimes causes color dispersion problems displaying different color properties depending on the viewing angle [39].

Despite all these previous efforts, it is not easy to tune the optical properties of the QD-adopted lighting once the QD is applied and set up completely in the lighting fixture. Once the QD component is set up in the lighting fixture, for example, QD films, the luminous flux, and the color properties are fixed and exhibit very slow changes over the usage time. Improving the flexibility of the QD-based lightings is a prerequisite for expanding the application of QD in the lighting area. Our previous simulation study revealed that one possible solution may be to adopt a QD cap structure in conventional LED lightings [40]. The combination of the red QD caps and commercially available lightings could be adopted to improve the color-rendering property substantially. The purpose of the present study is to suggest a new type of QD component—so-called QD caps—experimentally, which can be incorporated easily in a commercial white LED lighting fixture. The remote design was adopted for the long-term stability of QD-adopted lighting. This research demonstrates that the emitting properties, especially the color properties, of the LED lighting can be controlled in a wide range by adopting the present QD caps.

## 2. Materials and Methods

**Figure 1:**
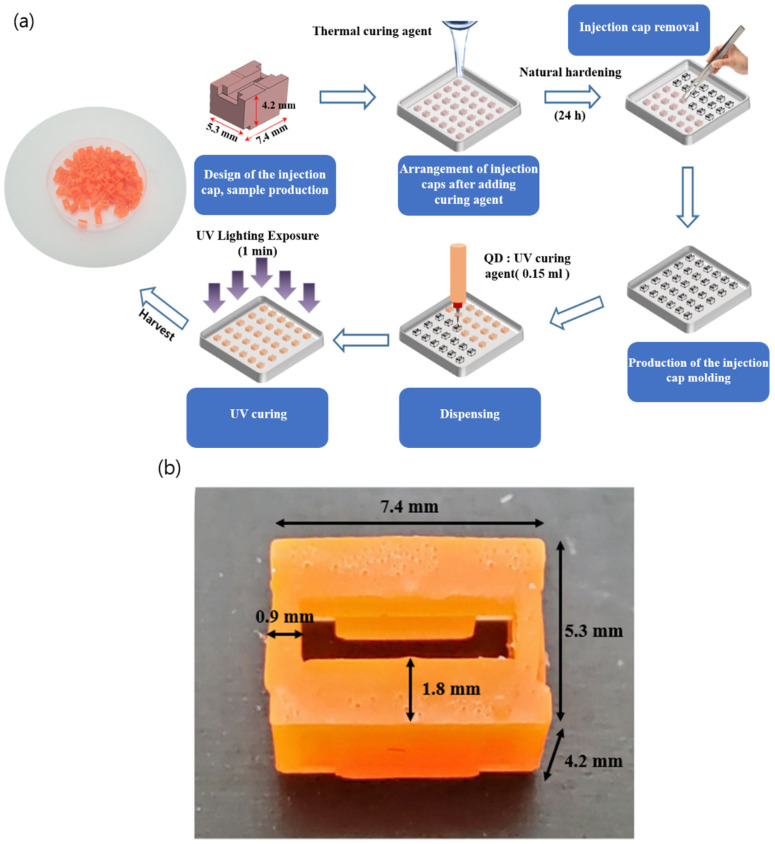
A schematic process of fabrication of red QD caps (**a**) and a picture of the fabricated red QD cap (**b**).

Figure 1a shows the fabrication process of the QD cap. First, the injection molding was designed based on the simulation results described in the next section and the required dimensions for white LED packages over which the QD cap will be placed. Polycarbonate was used to make sample QD caps, which were arranged periodically on the petri dish with an area of 100 × 100 mm^2^. The fabricated QD cap has outer dimensions of 7.4 × 5.3 × 4.2 mm^3^ and two lateral thicknesses of 0.9 and 1.8 mm. The upper surface has a rectangular opening with an area of 5.6 × 1.7 mm^2^. The QD was a red CdSe/ZnS with a core/shell structure and an average size of ~6 nm, which was mixed with irregular hollow silica (SG-HS40, Sukgyung AT Co., Ansan, Korea) for homogeneous dispersion. The shape of the hollow silica was irregular with an approximate average size of 40 ± 10 nm, a BET (Brunaue–Emmett–Teller) area of 400–500 m^2^/g, and a density of 2 g/cm^3^. In addition to the homogeneous dispersion, silica is known to be stable against moisture and air, which enhances the long-term stability of QDs. The details of the preparation of QD particles were described elsewhere [39].

The PDMS (Polydimethylsiloxane) hardener with the ratio of 10:1 between the base material and the curing agent was poured in the petri dish on which the cap samples were periodically arranged. After hardening for 24 h under ambient conditions, the cap samples were removed. The UV curing agent (Miracle UV Resin) and red CdSe/ZnS QD particles were mixed at an appropriate ratio and then poured (0.15 mL) into the mold by using the dispensing equipment (Super Sigma CMIII-V5, Musashi Engineering Inc., Tokyo, Japan). The QD cap was irradiated at the irradiance of 50 mW/cm^2^ for one min. by using the UV curing system (MSUV-L400L, MS Tech Co., Hwaseong, Korea), which hardened the QD cap. Due to the different functional groups of the PDMS mold and the urethane acrylate UV resin, the QD cap could be easily detached from the mold. Figure 1b shows the photo of the fabricated QD cap together with the dimensions.

**Figure 2:**
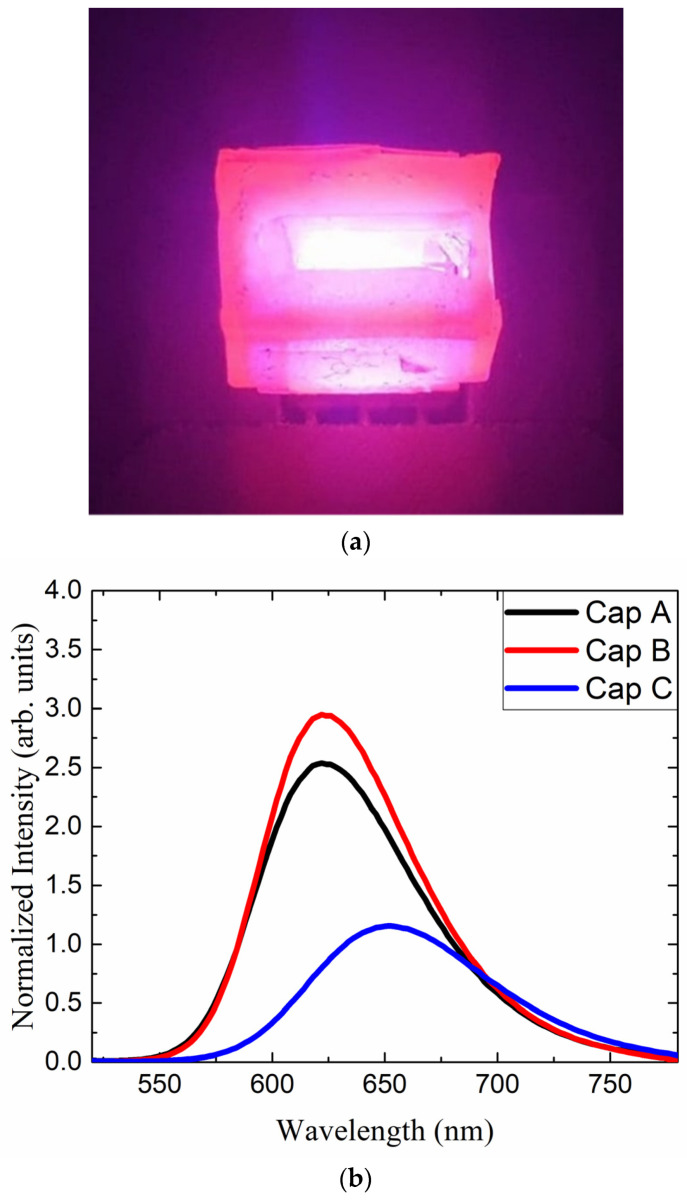
The photo of the emitting red QD combined with blue LED chips (**a**) and the photoluminescence emitting spectra of three kinds of red QD caps excited by blue LEDs at ~453 nm (**b**).

Two QD caps have been fabricated with the center wavelengths of 623 (denoted as QD cap <A>) and 652 nm (denoted as QD cap <C>) with a QD concentration of 5 wt%. In the case of the QD caps with 623 nm, additional QD caps with a concentration of 7.5 wt% were fabricated (which will be denoted as QD cap <B>). Figure 2a shows the picture of the emitting QD cap <A>, which was placed over a blue LED (IWS-L5056-UB-K3, Itswell Co. Ltd., Incheon, Korea). The overall color is purple due to the overlap of the blue and the red light. Figure 2b shows the emitting spectra of the three QD caps excited by the same blue LED. The spectra were normalized with respect to the blue peak. The emitting spectrum of the QD cap shows a slight red-shift when the concentration increases from 5 to 7.5%, which is attributed to the higher reabsorption at a larger concentration and is known as fluorescent quenching. The QD cap <C> shows a rather broad emitting spectrum at longer wavelengths.

**Figure 3:**
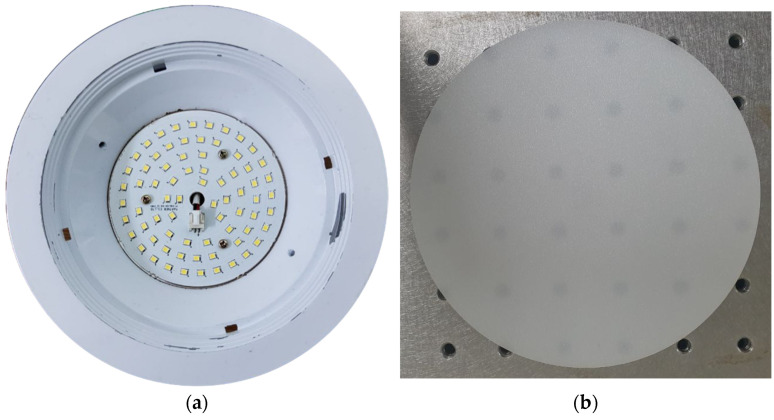
The photos of (**a**) the white LED lighting fixture and (**b**) the PC diffuser plate.

These QD caps were combined with a commercially available 6-inch, 15-W white LED lighting (KE15DN61S57A1, Partner Co., Gimpo, Korea). It consists of 72 white LEDs with an emitting area of 3.2 × 2.8 mm^2^ arranged concentrically. The lower and upper diameters of the lighting frame were 97 and 184 mm, respectively. The inclination angle of the side reflector was 131.5° with a reflectance of 76%. The reflectance of the PCB (printed circuit board) on which white LEDs were located was 69%. The luminous efficiency of the lighting is 105 lm/W (the total luminous flux is 1575 lm), and the color rendering index (CRI) is 83.1 with a correlated color temperature (CCT) of 5530 K. Figure 3a shows a photo of the white LED lighting fixture used in this study. In general, white LEDs form bright spots, which are the cause of glare phenomena, on the emitting plane; thus, a diffuser plate is necessary for removing them. A polycarbonate (PC) diffuser plate with a radius of 147 mm and a thickness of 2 mm was used, as shown in Figure 3b. The total transmittance and a haze of the diffuser plate were measured to be 56.48% and 99.45%, respectively. The haze property was measured by using a haze meter (NDH-2000N, Nippon Denshoku, Tokyo, Japan). The distance between the PCB and the bottom surface of the diffuser plate was 32 mm. Detailed dimensions and optical properties are summarized in Table 1.

**Figure 4:**
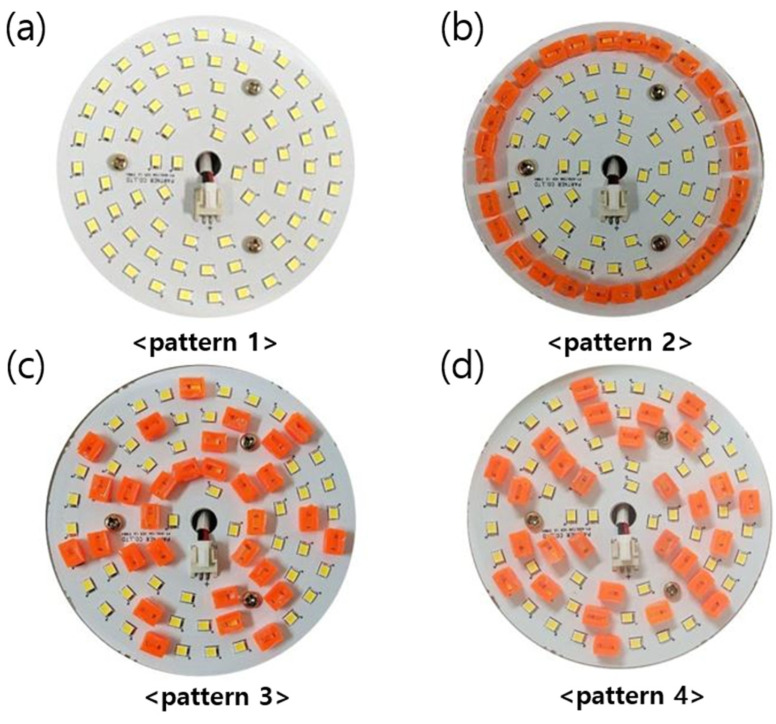
The photos of (**a**) the white LEDs arranged on the PCB and (**b**–**d**) three different arrangements of the red QD caps placed over the white LEDs.

Figure 4a shows the photo of the arrangement of white LEDs of the used lighting. Figure 4b–d display three patterns of the red QD caps on the lighting. The emitting spectrum, the luminance, and the color coordinates were recorded in terms of a spectroradiometer (PR670, PhotoResearch Co., Chatsworth, CA, USA). Both positional and angular dependences of these properties were investigated. The CRI and the illuminance were measured using an illuminance meter (SPIC-200A, Everfine, Hangzhou, China).

## 3. Results and Discussion

**Figure 5:**
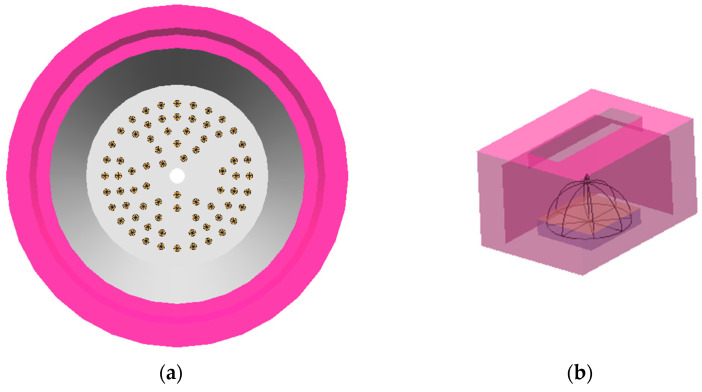
Simulation models of (**a**) the lighting fixture and (**b**) the red QD cap.

First, we describe the reason why the open cap structure was adopted for the QD cap design. The critical point is whether the opening window on the upper surface of the cap is necessary or not. To design the shape parameters of the QD cap, optical software (LightTools ver.9, Synopsis, Mountain View, CA, USA) was used to carry out the ray-tracing simulation. All dimensions of the white LED lighting used in the experiment were included in the simulation. Figure 5a,b show the simulation model of the lighting and the QD cap, respectively. The dimensions of the open cap were the same as those of the fabricated QD cap. An additional QD cap without any opening window was prepared for comparison. The host material of the cap was set to be PDMS. The intensity distribution of the white LED was set to be Lambertian. The diffuser plate was modeled by inserting TiO_2_ scatterers with an average radius of 220 nm with a Gaussian distribution into the PC material. The weight percent of TiO_2_ was 0.1 wt%, which was enough to prevent the formation of hot spots. The reported absorption spectrum and the quantum yield of the red QD were used for the simulation [41]. The measured emission spectra of two QD caps were also used in the simulation. These simulation conditions are shown in Supplementary Materials Figure S1a,b. All 72 LEDs were covered by the red QD caps in the simulation. The detailed simulation conditions are summarized in Supplementary Materials Tables S1 and S2.

**Figure 6:**
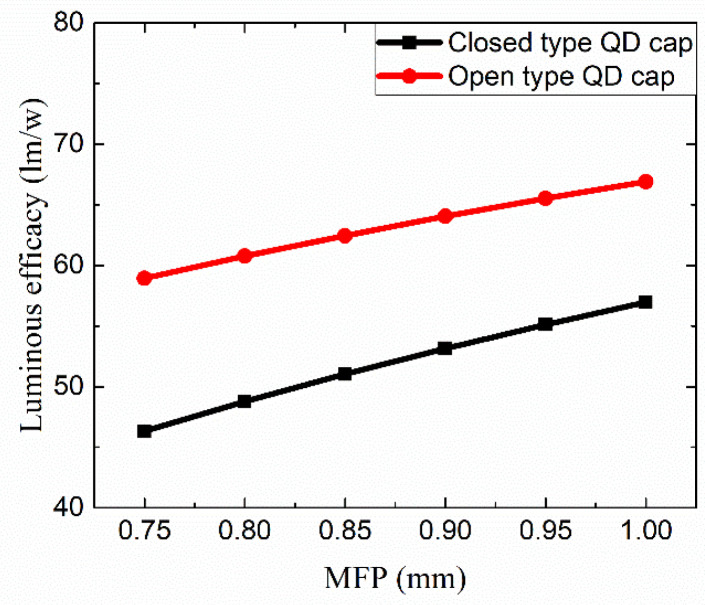
The dependence of the luminous efficacy as a function of the mean free path (MFP) of red QDs in the cap for the two designs, i.e., the open cap structure and the closed cap structure.

Figure 6 shows the dependence of the luminous efficiency as a function of the mean free path (MFP) of red QDs in the cap for the two designs, i.e., open cap and closed cap structures. This simulation result clearly shows that the efficiency of the lighting where open QD caps were applied is much higher than that where closed caps were adopted at the same MFP. The transmittance of the closed QD cap is lower than that of the open cap. Especially, the light generated from white LEDs may be trapped and absorbed in the closed QD cap at a higher probability because the light cannot escape directly from the inside cavity. Supplementary Materials Figure S2a,b show the dependence of the CCT and the CRI, respectively, on the MFP of red QDs included in the cap. The CCT of the lighting where closed QD caps were applied was higher than the other type under the same condition, which indicates that the color conversion efficiency via the closed-type QD cap is higher. This result is reasonable because the escaping light must pass through the QD cap, whereby part of the light would be converted into a red color in terms of the red QDs. On the other hand, the CRI of the open-type QD cap is higher than that of the closed-type QD cap under the same MFP. It may be attributed to the fact that the balance among all color components in the visible range becomes worse for the closed-type structure due to the strong enhancement of the deep red color. Considering the high efficiency and high CRI of the open-type QD cap, we decided to make a rectangular opening on the top of the red QD cap, as described in Section 2.

**Figure 7:**
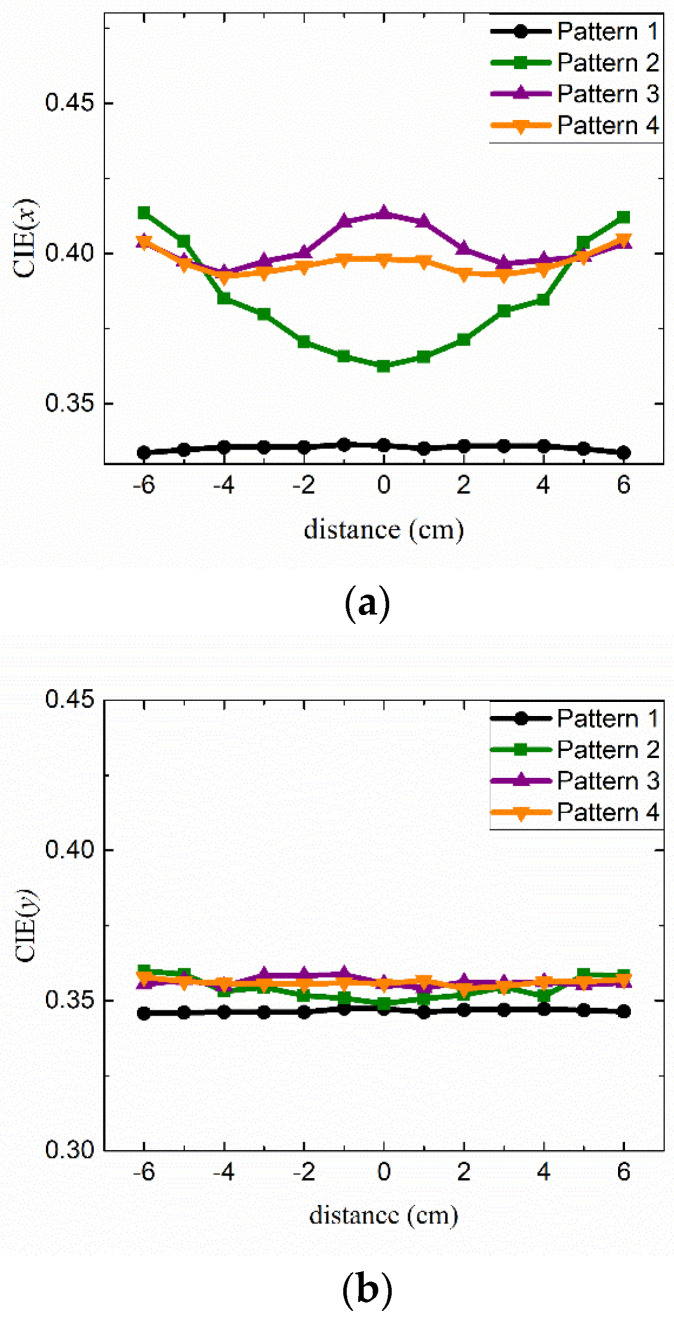
The positional dependence of the color coordinates (**a**) *x* and (**b**) *y* along the horizontal direction was measured for all four patterns shown in Figure 4.

Figure 7 shows the positional dependence of the color coordinates (*x, y*) displaced along the horizontal direction and measured for all four patterns shown in Figure 4. <Cap A> was used for all these measurements. Compared with pattern 1, which does not adopt any QD cap, all other patterns show larger color coordinates indicating efficient color conversion via red QD caps. Main changes in the color properties were associated with *x* because it is directly related to the relative portion of the red component in the whole visible range, which is enhanced by the red QD caps. The color coordinate *x* shows substantial changes depending on the measurement position for patterns 2 and 3, which is attributed to the inhomogeneous distributions of QD caps. Thus, we focus on the optical properties of the QD lighting with pattern 4, which shows the highest color uniformity. For example, the *x* values near the outer rim were significantly higher for pattern 2 where the QD caps were arranged near the circumference. Other patterns were also investigated resulting in no further improvement compared with pattern 4. Figure S3 in the Supplementary Materials shows the positional dependence of the color coordinates (*x, y*) of the QD lighting adopting pattern 4 along the horizontal (0°), diagonal (45°), and vertical (90°) directions, which exhibit nearly the same positional dependences. The standard deviations of *x* and *y* are approximately 0.005 and 0.001, respectively, in these cases.

**Figure 8:**
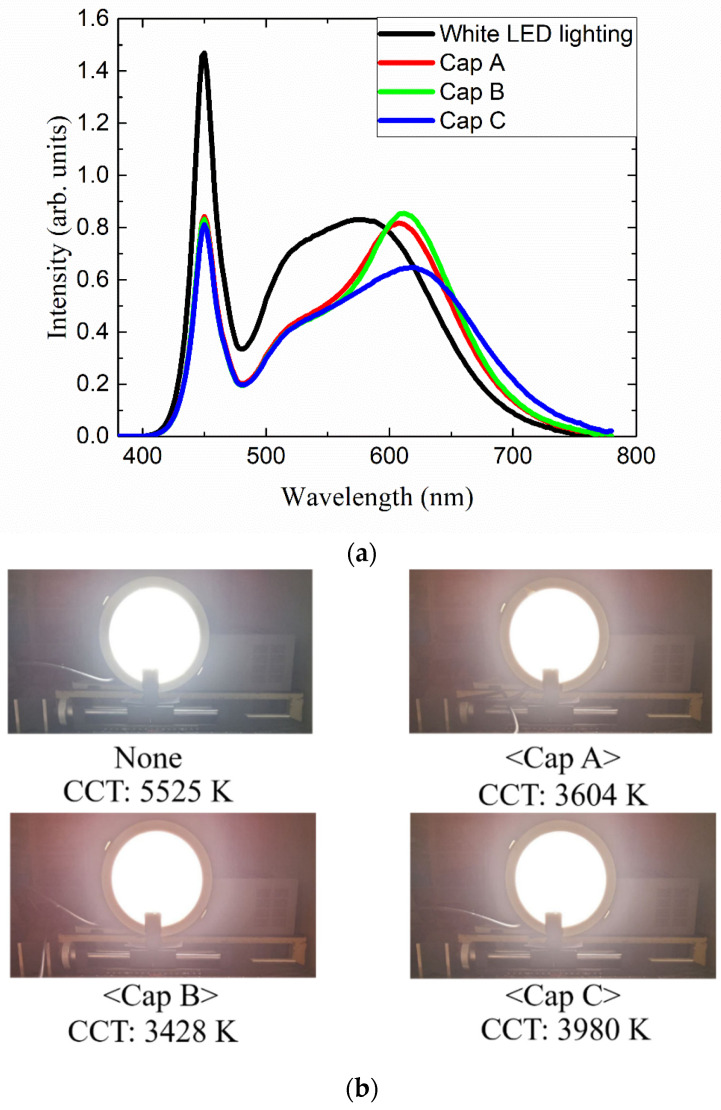
(**a**) Emitting spectra of the QD lighting adopting pattern 4 where three kinds of red QD caps have been used. “White LED lighting” indicates the original LED lighting where no QD cap was adopted. (**b**) The photographs of four LED lights demonstrating the change in color shade.

**Figure 9:**
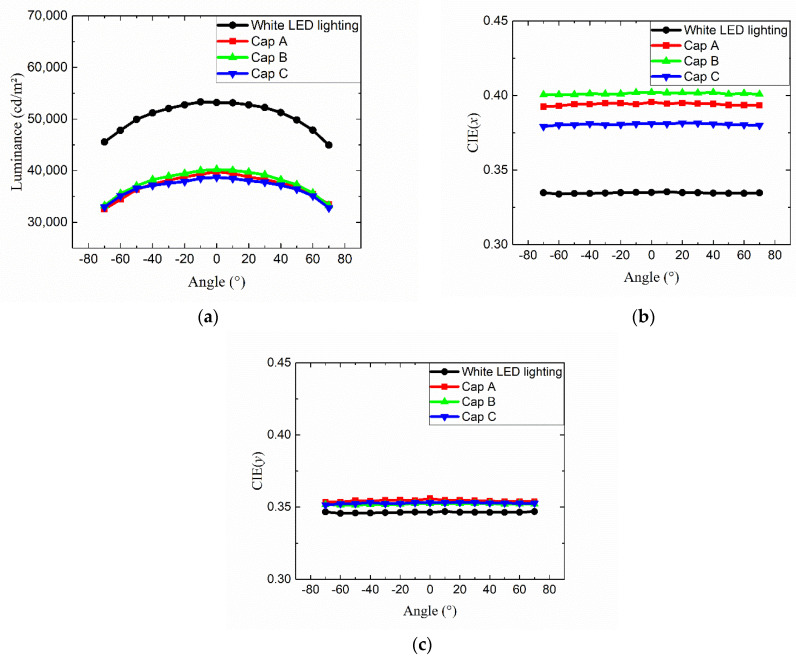
Angular dependence of (**a**) the luminance and the color coordinates (**b**) *x* and (**c**) *y* for the four configurations where no cap and cap A, B, and C were used.

Figure 8a shows the emitting spectra of the QD lighting adopting pattern 4, where three kinds of red QD caps have been used. Without any QD cap, the spectrum consists of a blue peak coming from the blue LED and a broad yellow peak emitted from the yellow phosphor layer coated over the LED chips. The addition of red QD caps enhances the intensity of the longer-wavelength region by forming a red peak in the 620–640 nm range. This color conversion process reduces the heights of the blue and green peaks. The overall shapes of the red peaks are nearly the same as the PL spectra shown in Figure 2b. Figure 8b shows photographs of four LED lightings demonstrating different color shades depending on the QD cap. Figure 9a displays the angular dependence of the luminance for the four configurations described above. The luminance is the largest along the on-axis direction and decreases mildly as the viewing angle increases. The luminance of the cap-adopted LED lighting is smaller than that of the original LED lighting without any QD cap by ~27%. This is mainly due to the reduced intensity in the green portion, which contributes to the luminous flux most significantly. Figure 9b,c show the angular dependence of the color coordinates *x* and *y*, respectively, which exhibit nearly no angular dependence. This indicates that the diffuser plate mixes the light enough to remove any possible difference in the optical path length depending on the viewing angle via the red QD cap. This result contrasts with the case where red QD films are adopted on the diffuser plate in the direct-lit white LED lighting [39]. When the QD film is on the diffuser plate, the optical path length in the film depends on the propagation angle of the excitation light, resulting in significantly higher color conversion at larger viewing angles. This kind of color dispersion problem is absent in the present QD-cap design.

**Figure 10:**
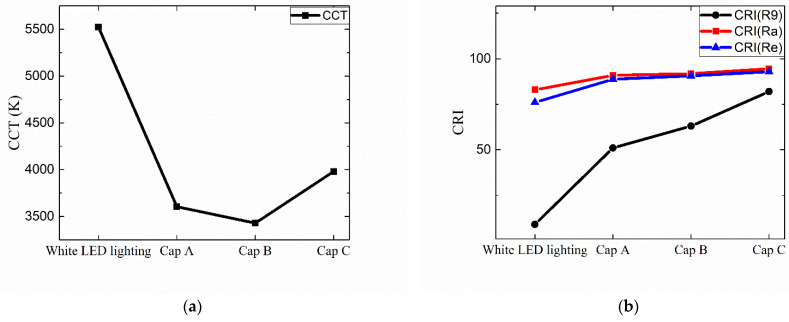
The dependence of (**a**) the CCT and (**b**) the three CRI values (Ra, Re, R9) on the optical configuration of the lighting.

Figure 10a,b show the CCT and the CRI of the four configurations. The CCT drops from ~5550 K to 3500–4000 K depending on which QD cap we adopt. The substantial change in the CCT is mainly due to the enhanced color conversion from blue/green to the red light via red QD caps. The *R9*, which is related to the deep red component, increases significantly when QD caps are adopted, as can be seen in Figure 8a, thanks to which the *Ra* and *Re* increase as well. Both *Ra* and *Re* are higher than 90 except for the case of <Cap A> where the *Re* is approximately 89. These values are a significant improvement compared with, for example, *Ra*~82.9 of the original white LED lighting without any red QD cap. These results clearly show that the adoption of red QD caps is an effective way to change the CCT in a wide range and to increase the color-rendering properties of conventional white LED lighting. The CRI of the lighting with <Cap C> is the highest, which is attributed to the broad and rather even distribution of spectral components in the visible range, as can be seen in Figure 8a.

**Figure 11:**
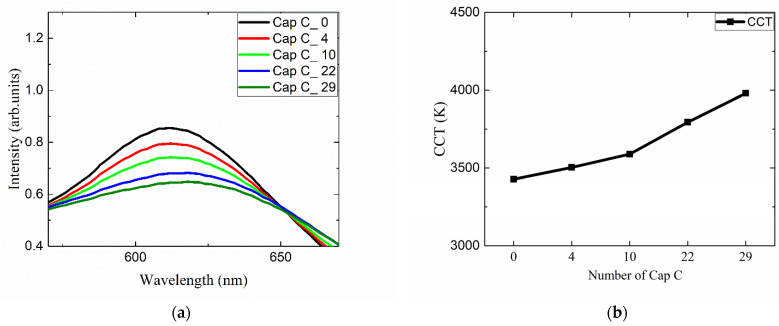
The dependence of (**a**) the emitting spectrum in the red region and (**b**) the CCT on the number of replaced caps.

**Figure 12:**
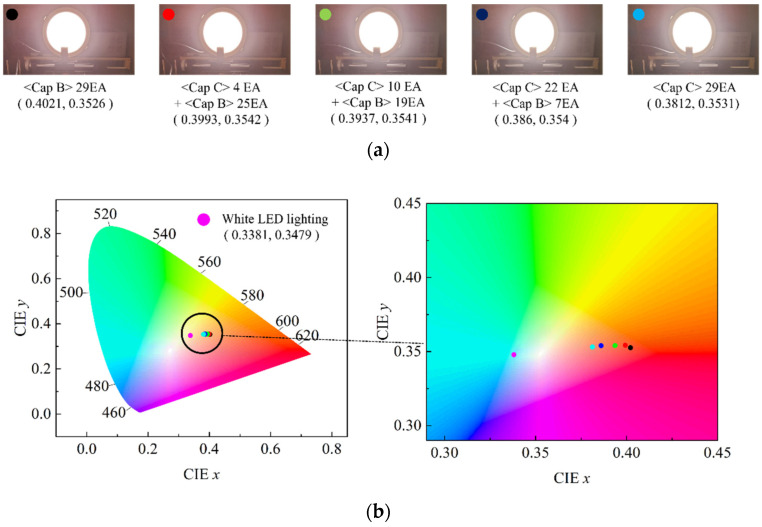
The change in the color shade of the white LED lighting depending on the QD cap configuration (**a**) and the change in the color coordinates on the chromaticity diagram (**b**). The red point in (**b**) denotes the original color coordinates with no QD caps being adopted.

The present study suggests that the color properties including the CCT and the CRI can be tuned in a wide range by adopting red QD caps. This modification can be easily performed by just placing an appropriate number of QD caps over the conventional white LEDs. The shape of the QD cap can be modified flexibly for various conventional LED lighting fixtures when the approach shown in Figure 1 is adopted. More functionalities can be attained by mixing different QD caps. To demonstrate this possibility, the QD <Cap B> were arranged in pattern 4, and then, part of the QD caps were replaced by <Cap C> in a concentric manner starting from the center of the lighting. The number of replaced QD caps was 0, 4, 10, 22, and 29. Supplementary Materials Figure S4a,b show the positional dependence of the color coordinates (*x, y*) along the horizontal direction measured for all cases. The standard deviations of *x* and *y* are approximately 0.005 and 0.001, respectively. The angular dependence of the color properties was investigated as well and there was no color dispersion for all cases, i.e., the color coordinates were nearly the same irrespective of the viewing angle. Figure 11a,b display the dependence of the emitting spectrum in the red region and the CCT on the number of replaced caps, respectively. As the number of <Cap C> increases, the peak height at ~610 nm decreases and the peak becomes broadened. As a result, the portion of the red component among the whole visible spectrum decreases, resulting in the increase in CCT. Figure 11b indicates that the CCT changes from ~3450 K to ~4000 K as the number of <Cap C> increases. Figure 12a shows the change in the color shade of all investigated LED lightings with different QD cap configurations. The corresponding change in the color coordinates on the CIE1931 chromaticity diagram is shown in Figure 12b as well. Compared with the original color coordinates where no QD caps were used (the red point on the chromaticity diagram), they shift to the right direction, i.e., larger *x* values when the number of replaced QD caps increases. The overall change is not large, but it can be tuned easily by adopting different QD caps having different spectral features.

The most noteworthy advantage of applying the red QD caps is the large tunability of the color property in a wide CCT range. Figure 10a shows that the CCT of ~5500 K of the original lighting was drastically reduced to ~3500 K by adopting the red QD caps. In addition, fine-tuning of CCT is also possible by mixing different QD caps. This is a noticeable advantage because a simple change of the red QD cap controls the color properties of the lighting in a wide CCT range. Especially, adopting red QD caps is an effective way to realize warm white shade below 4000 K. Another advantage of applying QD caps compared with applying the conventional remote QD film is that QD caps are incorporated directly over the LEDs. Thus, this design does not induce any serious problem caused by optical path length difference, which usually induces color dispersion depending on the viewing angle. A final advantage of this approach is that the long-term reliability of QD materials is expected due to the remote design. A simple coating of QDs on the hot LED chips induces degradation of the quantum efficiency of QDs, which is avoided in the present design.

Finally, we discuss the aging properties of the QD cap-applied white LEDs. Figure S5 in the Supplementary Materials shows the dependence of the CRI (Ra) on the aging time within 1500 h for the LED lighting where 12 QD caps are adopted. It clearly shows that the CRI does not change appreciably with time demonstrating the color stability of the present design. The luminous efficacy changed from 69.4 lm/W to 68.2 lm/W with a 1.7% decrease within the same time window. The color coordinates and CCT were also stable. These results indicate that the present remote design provides long-term color stability without any noticeable degradation over time.

## 4. Conclusions

A conventional white LED lighting fixture consisting of blue LED chips and yellow phosphor materials suffers from insufficient deep red component resulting in low color-rendering properties. To overcome this problem, a new concept of remote-type QD components is suggested in this study. Rectangular-shaped QD caps with an appropriate opening window were fabricated using a typical injection molding technique based on CdSe/ZnS QDs with a core/shell structure, PDMS soft molding, and UV curing agent together with hollow silica for homogeneous dispersion. The application of QD caps to a conventional white LED downlighting clearly showed a substantial increase in the color rendering index from 82.9 to 94.5 and significant controllability of the correlated color temperature between 5525 to 3428 K thanks to the easy tunability secured by adjusting the number of QD caps and their emitting spectra. Even though the CRI values of the present design are comparable to those of other QD-based lightings such as QD film-adopted LEDs, the present study revealed that QD caps are superior to other designs due to the negligible color dispersion, which is attributed to the rectangular QD cap that surrounds each LED, resulting in nearly no dependence of color properties on the optical path length difference. The newly suggested QD-cap-based LED lighting provides a very flexible way to control the color properties of commercially available white LED lighting by simply assembling appropriate red QD caps over the white LEDs.

## Figures and Tables

**Table 1:** Dimensions and optical properties of the lighting frame and the PC diffuser plate.

	Lighting Frame		Polycarbonate (PC) Diffuser Plate
Diameter (mm)	184 (upper)	Diameter (mm)	147
Height (mm)	32	Thickness (mm)	2
PCB board diameter (mm)	97	Transmittance (%)	56.48
Reflectance	Inner side reflectance: 76% Inclination angle of the inner side: 131.5° PCB board reflectance: 69%	Haze (%)	99.45
		Parallel transmittance (%)	0.31
		Diffuse transmittance (%)	56.17

## Data Availability

Data presented in this article is available on request from the corresponding author.

## Supplementary Materials

The following are available online at https://www.mdpi.com/article/10.3390/nano12071097/s1, Figure S1: Simulation conditions, Figure S2: Simulation results, Figure S3: Positional dependence of color coordinates along different directions, Figure S4: Positional dependence of color coordinates for different configurations, Figure S5: Aging property of the CRI, Table S1: Simulation conditions, Table S2: Simulation conditions.
