# Supplementary material for: Substantial Improvement of Color-Rendering Properties of Conventional White LEDs Using Remote-Type Red Quantum-Dot Caps
Source: Nanomaterials (Basel). 2022 Mar 27;12(7):1097. doi: 10.3390/nano12071097 (PMC9000883; doi:10.3390/nano12071097)
Supplement: Supplementary file 1 [file nanomaterials-12-01097-s001.zip › nanomaterials-1592111-supplementary.pdf]

nanomaterials

Supplementary Materials

Substantial Improvement of Color-Rendering Properties of Conventional White LEDs Using Remote-Type Red Quantum-Dot Caps

Gi Jung Lee [1], Seung Chan Hong [1], Jung-Gyun Lee [1], Jae-Hyeon Ko [1,*], Taehee Park [2], Young Wook Ko [2], and Sergey Lushnikov [3]

Table S1. Simulation parameters of the QD-applied white LED lighting.

	**Lighting Frame**	**Diffuser plate**	**Quantum dot cap**
Diameter (mm)	182	146	Outer wall: 7.4 × 5.3 × 4.2<br>Attachment side: 5.6 × 3.4<br>Opening side: 5.6 × 1.7
Thickness (mm)	28 (Height)	2	Attachment side: 1.8 × 0.9<br>Opening side: 1.8 × 1.2
Material	PMMA (n = 1.4936)	Polycarbonate &<br>TiO_2<br>(n = 2.4358)	PDMS&<br>Red quantum dot (n = 1.43)
Optical property	Inner side reflectance: 76%<br>Inclination angle of the inner side: 126°<br>PCB board reflectance: 69%	Optical smoothing<br>(Fresnel loss)	Optical smoothing<br>(Fresnel loss)

Table S2. Simulation parameters of white LEDs included in the lighting model.

	**White LEDs**				
Array radius (mm)	10	17.1	24.2	31.3	38.4
Number of LEDs	3	8	12	21	28
Radiant Flux (w) per LED	0.5 (154.25 lm)				
LED dimensions (mm)	2.8 (length) × 3.2 (width) × 0.7(height)				
CRI (Ra)	82.9				
CCT(k)	5382				

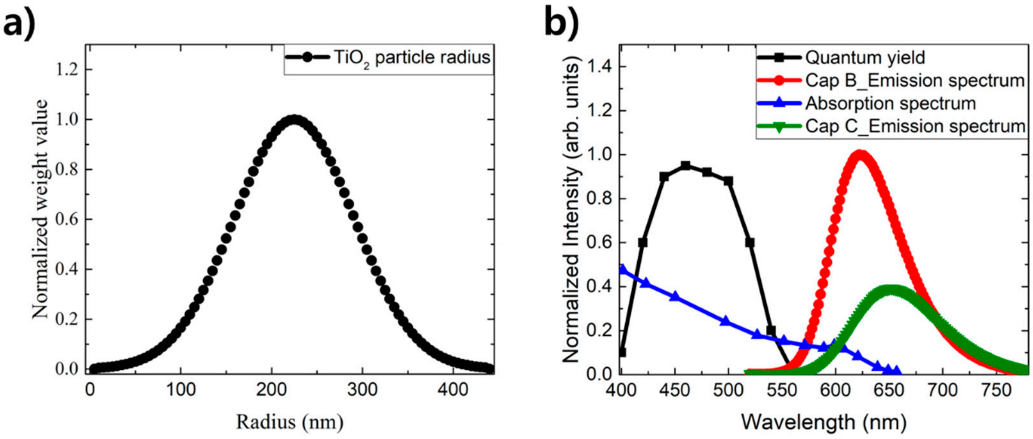

Figure S1. Simulation conditions: (**a**) the size distribution of the TiO_2 particles in the diffuser plate, and (**b**) the quantum yield, the absorption spectrum and two emission spectra.

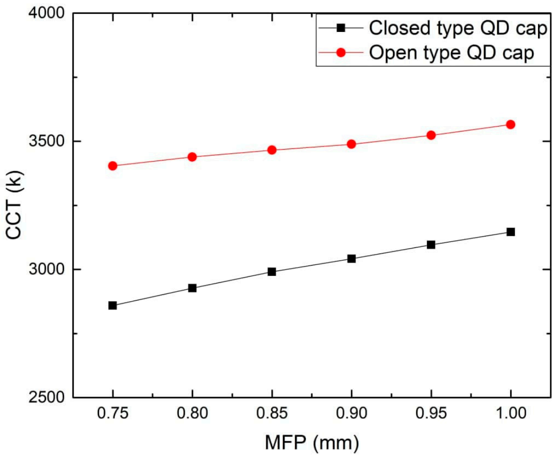

(**a**)

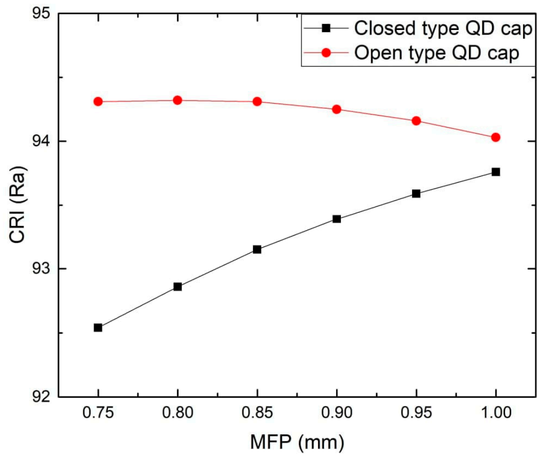

(**b**)

Figure S2. Simulation results for the dependence of (**a**) the CCT and (**b**) the CRI on the MFP of red QDs in the cap.

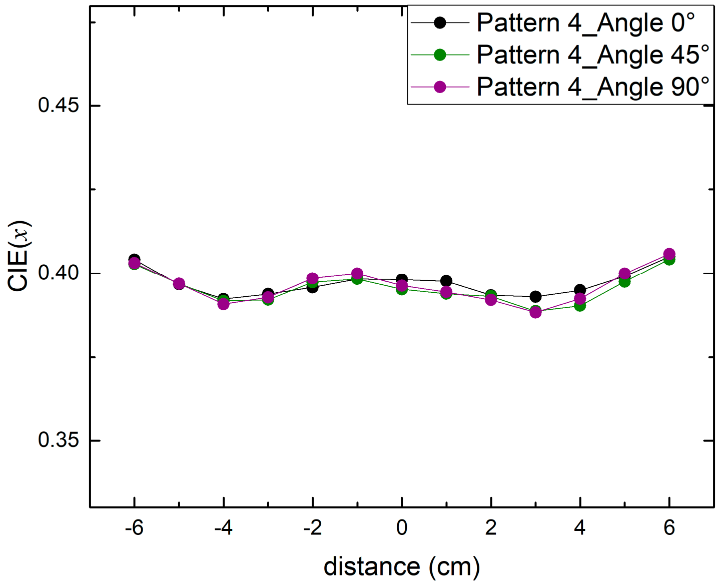

(**a**)

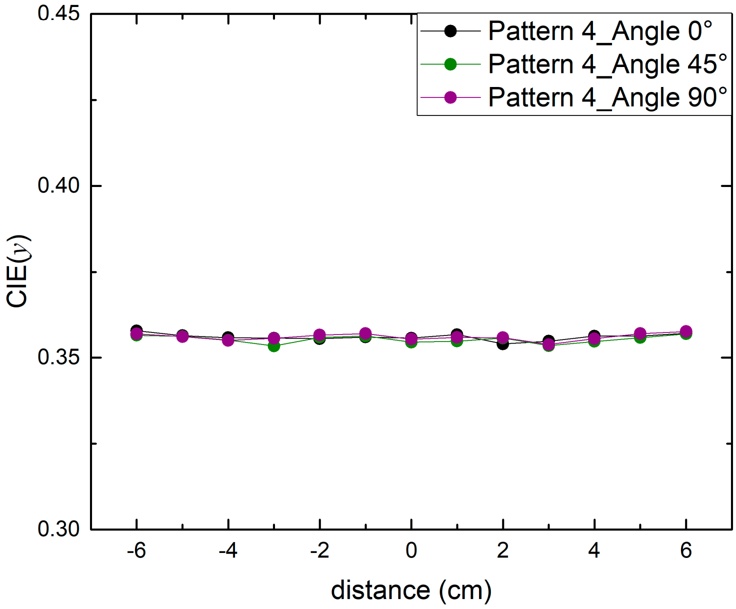

(**b**)

Figure S3. The positional dependence of the color coordinates (x, y) of the QD lighting adopting the pattern 4 measured along the horizontal (0°), diagonal (45°), and vertical (90°) directions. The sub-figures (**a**) and (**b**) are for the color coordinates x and y, respectively.

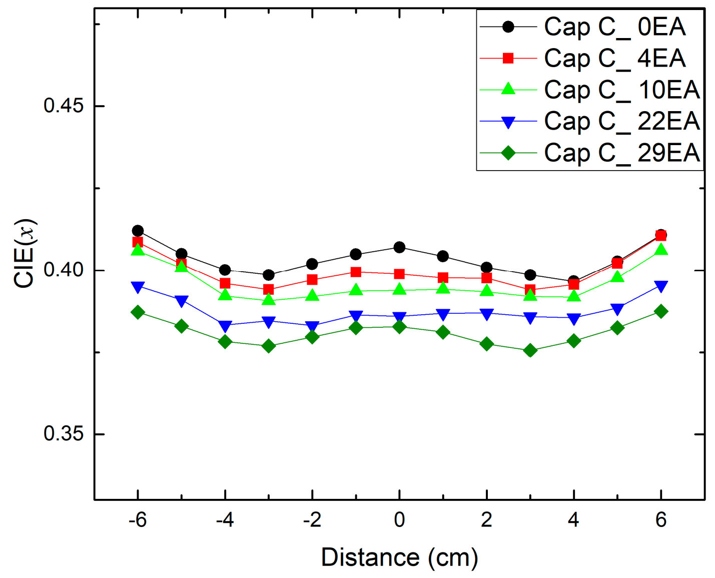

(**a**)

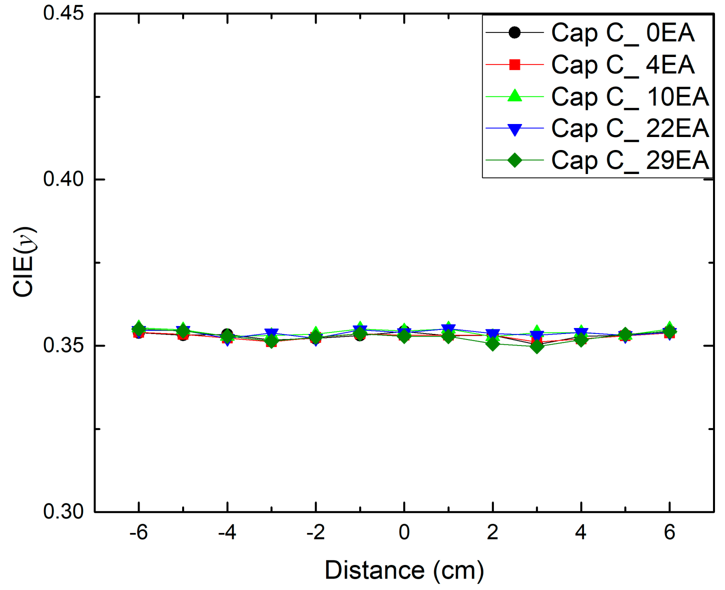

(**b**)

Figure S4. The positional dependence of the color coordinates (x, y) of the QD lighting for all investigated configurations along the horizontal direction. The subfigures (**a**) and (**b**) are for the color coordinates x and y, respectively.

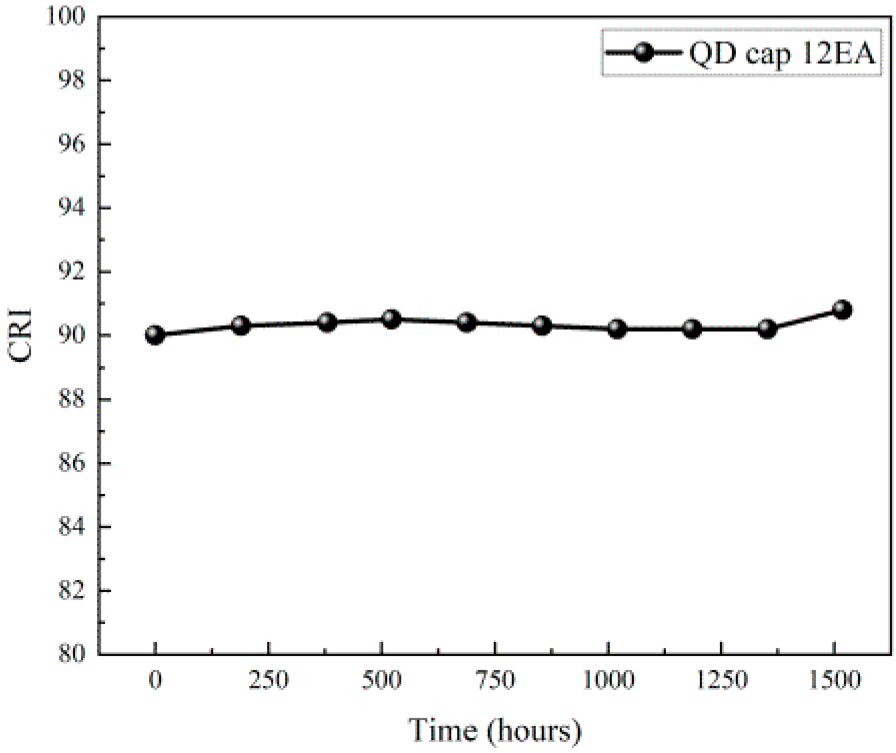

Figure S5. The dependence of CRI on the aging time.
